# Long-term alterations of striatal parvalbumin interneurons in a rat model of early exposure to alcohol

**DOI:** 10.1186/1866-1955-4-18

**Published:** 2012-07-03

**Authors:** Andrea De Giorgio, Sara E Comparini, Francesca Sangiuliano Intra, Alberto Granato

**Affiliations:** 1 Department of Psychology, Catholic University, L.go A. Gemelli 1, Milan 20123, Italy

**Keywords:** Fetal alcohol, GABA, Interneuron, Parvalbumin, Striatum, Voronoi tessellation

## Abstract

**Background:**

Exposure to alcohol *in utero* is a known cause of mental retardation. Although a certain degree of motor impairment is always associated with fetal alcohol spectrum disorder, little is known about the neurobiological basis of the defective motor control. We have studied the striatal interneurons containing parvalbumin in a rat model of fetal alcohol spectrum disorder.

**Methods:**

Newborn rats received ethanol by inhalation from postnatal day two through six and parvalbumin striatal neurons were labeled by immunohistochemistry on postnatal day 60. The spatial distribution of parvalbumin interneurons was studied using Voronoi spatial tessellation and their dendritic trees were completely reconstructed.

**Results:**

Parvalbumin interneurons of ethanol-treated animals showed a clustered spatial distribution similar to that observed in control animals. The dendritic tree of parvalbumin interneurons was significantly reduced in ethanol-treated animals, as compared with controls.

**Conclusions:**

Striatal parvalbumin interneurons are crucial components of the brain network serving motor control. Therefore, the shrinkage of their dendrites could contribute to the motor and cognitive symptoms observed in fetal alcohol spectrum disorder.

## Background

Fetal alcohol spectrum disorder (FASD) represents one of the leading causes of mental retardation in western countries [1]. FASD is characterized by multiple impairments such as deficit of attention and learning, language difficulties, and mnemonic and intellectual development anomalies [2]. Its genesis can be explained by the deep structural and functional alterations of cortical neurons [3,4]. Serious motor impairments have also been described [5] and can be partly explained by the altered functional organization of the motor cortex [6,7]. However, motor planning and execution are complex tasks, which also involve the basal ganglia [8]. Gamma-aminobutyric acid (GABA) interneurons expressing parvalbumin (PV), though quantitatively not prominent, play a key functional role within the striatum. PV interneurons display convergent input from different cortical areas [9] and in the human striatum they are preferentially distributed in the motor territory [10]. In addition, it has been demonstrated that the fetal migration of GABAergic interneurons from the medial ganglionic eminence to their final target is affected by alcohol [11]. Despite their known involvement in motor control and their possible susceptibility to early alcohol exposure, there are no studies addressing the anatomical organization of striatal PV interneurons after early exposure to alcohol. To this purpose, we carried out an immunohistochemical investigation of these neurons in adult rats exposed to ethanol inhalation during the first week of postnatal life. Owing to the immaturity of the rat brain at birth, this developmental stage represents the third trimester equivalent of human gestation [12]. Therefore, our experimental model resembles several aspects of human FASD (reviewed in [13]).

## Methods

All the experiments were conducted in accordance with the Society for Neuroscience Policies on the Use of Animals and Humans in Neuroscience Research.

Newborn Wistar rats (Et group) received ethanol by inhalation from the second postnatal day (P2; P0 is the birthdate) through P6. The method of ethanol administration has been described elsewhere [14]. Briefly, an air pump (air flow = 3 L/min) was connected to a vaporization chamber into which ethanol (95% v/v) was injected at a rate of 2.5 mL/min. The ethanol vapors were conveyed to a Plexiglas cage in which the pups were placed, after separation from the mothers, for 3 h a day. This method of ethanol administration yields peak blood alcohol concentration ranging from 150 to 300 mg/dL [14,15]. Control animals of matched age (C group) were separated from the mothers for 3 h a day, omitting the ethanol inhalation procedure. Animals of both groups were weighed at P7, P15 and P60. No significant differences between the mean weight values of Et and C cases were observed at all the considered ages.

At P60, four male animals from the Et group and four from the C group were deeply anaesthetized (intraperitoneal injection of tiletamine/zolazepam (Zoletil) 10 mg/kg, and xylazine 10 mg/kg) and perfused through the ascending aorta with phosphate buffered saline followed by 4% buffered paraformaldehyde. The brains were cut on a freezing microtome into coronal sections 50 μm thick. Every fifth section through the caudate nucleus was processed for PV immunohistochemistry. Monoclonal antibodies were used for the detection of PV (Sigma, St Louis, MO, USA; P3088, dilution 1:5,000). The overnight incubation with primary antibodies was followed by a standard streptavidin-biotin immunoperoxidase procedure. Control sections were incubated in a medium lacking the primary antibody. The immunoreaction was revealed using the diaminobenzidine as a chromogen and a nickel intensification.

**Figure 1:**
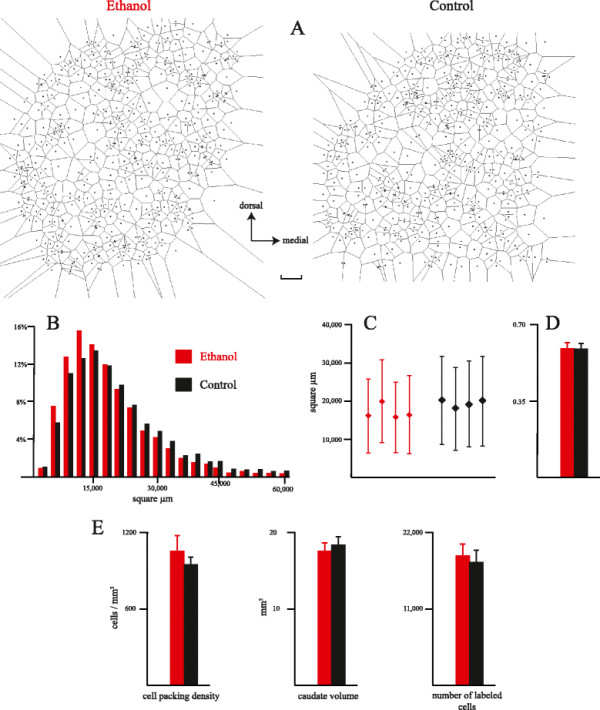
**Spatial distribution of striatal interneurons.** (**A**) Voronoi tessellation of two representative sections of the caudate nucleus from an ethanol-treated and a control case. Bar = 250 μm. (**B**) Frequency distribution of the Voronoi polygon areas in ethanol-treated and control cases. Polygon areas larger than 60,000 μm^2^ were excluded from the quantitative analysis. Bins = 3,000 μm^2^. (**C**) Mean and standard deviation of the polygon areas for each of the studied ethanol-treated and control cases (four sections per case). (**D**) Mean coefficient of variation, calculated as the ratio between the standard deviation and the mean of polygon areas. (**E**) Packing density of labeled neurons, total volume of caudate nucleus (from the rostral pole to the appearance of globus pallidus), and total number of labeled neurons in the caudate.

Sections were observed under a Nikon E 600 light microscope (Nikon Corporation, Tokyo, Japan). Immunoreactive striatal neurons have been charted with the aid of the software Neurolucida (Microbrightfield, Williston, VT, USA). The fine distribution of PV-immunoreactive (IR) neurons in the striatum was studied by means of Voronoi diagrams (Figure 1A), obtained using XYZ GeoBench for Macintosh (developed by Peter Schorn at ETH Zurich, Switzerland). Counts of labeled neuron profiles were corrected using Abercrombie’s method [16]. The volume of the caudate nucleus, from its rostral pole to the appearance of the globus pallidus, was estimated using Cavalieri’s principle [17].

Labeled neurons were chosen using a systematic random sampling of sections and their dendritic trees were reconstructed when at least 50% of their end points were contained in the section. The following quantitative parameters were evaluated: length of the dendritic tree of each neuron; length of each dendrite (provided that all the end points were contained in the section); number of primary dendrites for each neuron; number of end points of each dendrite; path distance, that is, the distance from the soma to each end point; terminal length percentage, that is, the percentage of the total dendritic length occupied by terminal branches. Data concerning the percentage of the image covered by immunostained somata and dendrites were obtained from randomly chosen frames (width: 640 μm; height: 380 μm), using the automatic threshold algorithm of ImageJ (http://rsb.info.nih.gov/ij/). Sholl analysis was carried out counting the intersections of dendrites with soma-centered circles of 10 μm, 20 μm, and 30 μm radius.

Unless otherwise indicated, all values are given as mean ± standard deviation and differences between groups were evaluated using the analysis of variance (ANOVA) for nested design.

## Results

Both in Et and C animals, PV-IR neurons were distributed throughout the caudate nucleus. Voronoi diagrams (Figure 1A), which delineate the polygon of free space surrounding each neuron, have proven useful to describe the spatial distribution of neurons both in normal and pathological conditions [18,19]. The distribution of polygon areas, shown in Figure 1B, was similar for Et and C animals, even though the Et distribution was slightly shifted to the left. The average polygon area was slightly smaller for Et than for C cases (Et = 17,026.03 ±1,966.24 μm^2^; C = 19,311.67 ±1,034.08 μm^2^; ANOVA for nested design: F_1,6_ = 4.23; *P* = 0.09). The standard deviation of the polygon area within each case (Figure 1C) is a measure of the tendency of neurons to be distributed into clusters (clustered distributions are characterized by high standard deviations [18]). As shown in Figure 1D, the coefficient of variation (the ratio between standard deviation and mean) was strictly similar in the two considered groups (about 0.60) and indicates that both in Et and C cases, PV-IR neurons are clustered. The packing density of labeled neurons, the volume of the caudate, and the total number of labeled cells did not show any significant difference between C and Et cases (Figure 1E).

**Figure 2:**
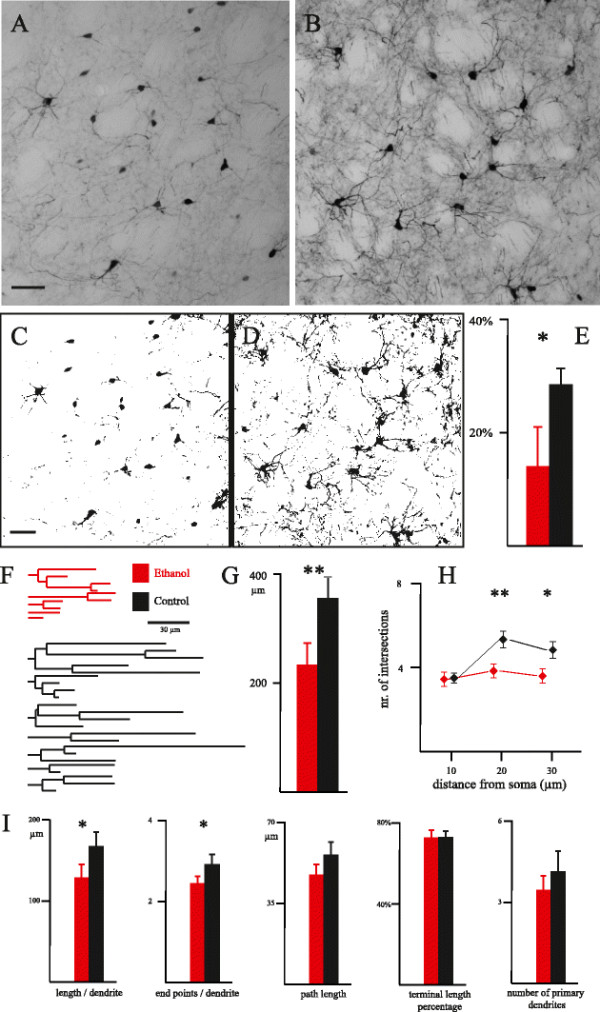
**Dendritic tree of striatal interneurons.** (**A**, **B**) Microphotographs showing the parvalbumin immunostaining of the caudate in two representative sections from an ethanol-treated (A) and a control (B) animal. Scale bar = 50 μm. (**C**, **D**) The same sections shown in A and B, after application of the threshold algorithm, in order to quantify the area covered by immunostaining. Scale bar = 50 μm. (**E**) Average percentage of the image area covered by immunostaining (two frames per section; width: 640 μm; height: 380 μm; four sections per case). * *P* <0.05 at the ANOVA for nested design. (**F**) Dendrograms of two representative neurons, from an ethanol-treated and a control animal. (**G**) Average length of the dendritic tree of each neuron (five neurons per section; four sections per case). **P <0.01 at the ANOVA for nested design. (**H**) Sholl analysis performed with circles of 10 μm, 20 μm and 30 μm radius. Error bars represent standard error of the mean. Differences between the two groups were evaluated with the Mann–Whitney *U* test (*P < 0.05; **p <0.01). (**I**) Bar graphs showing quantitative parameters for completely reconstructed dendrites. The average number of primary dendrites per neuron is represented in the last bar graph. *P <0.05.

In all the studied cases, PV-IR neurons showed a medium-sized cell body, with two to eight aspiny varicose dendrites emerging from the soma (Figure 2A,B). Both in Et and C cases, cells and processes were heavily immunostained. In most cases, labeled dendrites, when contained completely within the considered section, displayed a regular tapering and could be followed up to their end points. In general, in Et animals the immunostained dendrites appeared less branched and shorter, as compared with control cases. As a consequence, the neuropil staining was much more intense in C than in Et animals (Figure 2A,B). We used two different methods to quantify this difference. Firstly, we used an automatic threshold algorithm followed by the measurement of the percentage of the image covered by immunostaining. As shown in Figure 2C-E, the average area with immunoreactivity was significantly larger in C than in Et cases (Et = 13.76 ±7.84%; C = 28.41 ±3.10%; F_1,6_ = 12.06, *P* <0.05). We also reconstructed the dendritic tree of randomly chosen PV-IR neurons (altogether, 80 Et neurons and 80 C neurons; Figure 2F,G). This procedure confirmed the above reported data, showing that the average length of the dendritic tree of single neurons was shorter in Et animals and the difference was highly significant (Et = 236.03 ±42.15 μm; C = 358.20 ±40.31 μm; F_1,6_ = 17.55, *P* <0.01). Furthermore, the complexity of the dendritic tree was also reduced in Et animals, as demonstrated by the Sholl analysis (Figure 2H), by a significantly lower length of single dendrites, and by a significantly lower number of end points per dendrite (Figure 2I). As shown in Figure 2I, a reduction of the path length also contributed to the shrinkage of the dendritic tree (at the edge of significance: F_1,6_ = 5.16; *P* = 0.06). The terminal length percentage and the number of primary dendrites were not significantly different in the two considered groups (Figure 2I).

## Discussion

The main finding of the present paper is the demonstration that early postnatal exposure to alcohol leads to long-term anomalies of striatal parvalbumin interneurons, mainly represented by the shrinkage of their dendritic tree. Data obtained from Voronoi tessellation show that PV interneurons of ethanol-treated animals maintain the same clustered spatial distribution as in normal animals.

Our data on the total number of PV-labeled neurons in the caudate argue against a selective susceptibility of striatal interneurons to alcohol-induced apoptosis [20]. This is in good agreement with our previous observation that the number of PV neocortical neurons is not reduced following early postnatal exposure to alcohol [15]. Interestingly, both striatal and neocortical PV interneurons share several features, such as the fast-spiking electrophysiological behavior [21,22] and an origin from the medial ganglionic eminence [23]. It appears that another common feature of PV interneurons is the relative resistance to alcohol-induced apoptotic death and/or migration defects. However, we cannot rule out alternative hypotheses. For instance, though undergoing apoptosis during ethanol exposure, striatal interneurons might still be generated and migrate to the striatum after ethanol withdrawal. In fact, postnatal neurogenesis of striatum-bound GABAergic interneurons has been demonstrated in the subventricular zone [24].

The simplified dendritic tree of striatal PV interneurons described in our study for ethanol-treated animals could be the consequence of the direct effect of alcohol during neuronal differentiation. It is known that alcohol interferes directly with dendritogenesis [25] through multiple mechanisms (discussed in [26]). However, it should be pointed out that ethanol could exert its effect through indirect mechanisms, since PV striatal interneurons are exquisitely susceptible to the modification of neural circuits in which they are involved. For instance, cerebellar damage is a well-documented feature of FASD [27] and it has been demonstrated that hemicerebellectomy leads to a progressive shrinkage of the dendritic tree of striatal interneurons [28]. Moreover, these neurons receive direct projection from corticostriatal neurons [29], mainly located in layer 5 [30]. We have recently demonstrated that the electrophysiological properties of layer 5 neurons are strongly impaired after early exposure to alcohol [4].

It is interesting to note that the reduction of the basal dendrites of neocortical pyramidal neurons after early exposure to alcohol is mainly due to a decreased dendritic branching rather than to a defect of elongation [26]. The reduction of end points observed in the present study suggests that the same mechanism is operant also for the striatal interneurons. However, a certain degree of defective elongation could also contribute, as suggested by the reduced path length (at the edge of significance).

Whatever the reason accounting for the structural anomalies of PV interneurons, the shrinkage of their dendritic trees is likely to have a deep functional impact on the function of the striatal circuitry. PV is able to modulate the amplitude of calcium transients in neurons and can therefore regulate synaptic plasticity [31]. In addition, PV fast-spiking interneurons of the striatum are extensively connected through dendritic gap junctions [32] and, similar to what happens in the neocortex, this feature enables them to mediate oscillatory synchronization within the gamma band [33]. As a consequence, they are thought to coordinate the spike timing of the medium spiny neurons, which represent the main output from the striatum [34]. Thus, the reduced PV dendritic network observed in our experimental model of FASD might impair the feed-forward striatal circuit and the striatal output as well.

## Conclusions

Although the cerebral cortex and cerebellum are usually considered the main structures targeted by early alcohol exposure, there is now increasing evidence that the basal ganglia are also deeply affected during FASD [35,36]. Our data provide further support to the idea that striatal alterations play a fundamental role in the genesis of symptoms associated with early alcohol exposure.

## Abbreviations

ANOVA, analysis of variance; C, control; Et, ethanol-treated; FASD, fetal alcohol spectrum disorder; GABA, gamma-aminobutyric acid; IR, immunoreactive; P, postnatal day; PV, parvalbumin.

## Competing interests

The authors declare that they have no competing interests.

## Authors’ contributions

ADG and AG designed the experiments, and performed the alcohol exposure and the immunohistochemistry. SEC and FSI collected the data. All authors analyzed the data. ADG and AG wrote the manuscript. All authors read and approved the final manuscript.
