# Erratum to: Long-term alterations of striatal parvalbumin interneurons in a rat model of early exposure to alcohol

**DOI:** 10.1186/s11689-016-9173-6

**Published:** 2016-10-24

**Authors:** Andrea De Giorgio, Sara E. Comparini, Francesca Sangiuliano Intra, Alberto Granato

**Affiliations:** Department of Psychology, Catholic University, L.go A. Gemelli 1, Milan, 20123 Italy

## Erratum

Upon publication of the original article [1], it was noticed that Francesca Sangiuliano Intra’s name displayed incorrectly in the article’s PubMed citation.

‘Francesca’ and ‘Sangiuliano’ were tagged in the article XML as ‘given names’ and ‘Intra’ as the ‘surname’, whereas ‘Francesca’ should be the only ‘first name’ and ‘Sangiuliano Intra’ the ‘surname’.

This means that the PubMed citation should be ‘Sangiuliano Intra F’ instead of ‘Intra FS’.

This has now been corrected in this erratum.
